# Development of a Machine Learning-Based Model to Predict Timed-Up-and-Go Test in Older Adults

**DOI:** 10.3390/geriatrics8050099

**Published:** 2023-10-07

**Authors:** Moritz Kraus, Ulla Cordula Stumpf, Alexander Martin Keppler, Carl Neuerburg, Wolfgang Böcker, Henning Wackerhage, Sebastian Felix Baumbach, Maximilian Michael Saller

**Affiliations:** 1 Department of Orthopedics and Trauma Surgery, Musculoskeletal University Center Munich, University Hospital of Ludwig-Maximilians-University (LMU), 81377 Munich, Germany; ulla.stumpf@med.uni-muenchen.de (U.C.S.); alexander.keppler@med.uni-muenchen.de (A.M.K.); carl.neuerburg@med.uni-muenchen.de (C.N.); wolfgang.boecker@med.uni-muenchen.de (W.B.); sebastian.baumbach@med.uni-muenchen.de (S.F.B.); maximilian.saller@med.uni-muenchen.de (M.M.S.); 2 Faculty of Sport and Health Sciences, Technical University of Munich, 80809 Munich, Germany; henning.wackerhage@tum.de

**Keywords:** frailty, clinical assessment, machine learning, TUG test, age, osteoporosis

## Abstract

Introduction: The measurement of physical frailty in elderly patients with orthopedic impairments remains a challenge due to its subjectivity, unreliability, time-consuming nature, and limited applicability to uninjured individuals. Our study aims to address this gap by developing objective, multifactorial machine models that do not rely on mobility data and subsequently validating their predictive capacity concerning the Timed-up-and-Go test (TUG test) in orthogeriatric patients. Methods: We utilized 67 multifactorial non-mobility parameters in a pre-processing phase, employing six feature selection algorithms. Subsequently, these parameters were used to train four distinct machine learning algorithms, including a generalized linear model, a support vector machine, a random forest algorithm, and an extreme gradient boost algorithm. The primary goal was to predict the time required for the TUG test without relying on mobility data. Results: The random forest algorithm yielded the most accurate estimations of the TUG test time. The best-performing algorithm demonstrated a mean absolute error of 2.7 s, while the worst-performing algorithm exhibited an error of 7.8 s. The methodology used for variable selection appeared to exert minimal influence on the overall performance. It is essential to highlight that all the employed algorithms tended to overestimate the time for quick patients and underestimate it for slower patients. Conclusion: Our findings demonstrate the feasibility of predicting the TUG test time using a machine learning model that does not depend on mobility data. This establishes a basis for identifying patients at risk automatically and objectively assessing the physical capacity of currently immobilized patients. Such advancements could significantly contribute to enhancing patient care and treatment planning in orthogeriatric settings.

## 1. Introduction

A key challenge in geriatric medicine is to develop objective measures that report a patient’s physical capability. Such biomarkers would help to base treatment decisions more on evidence. The Timed-Up-and-Go test (TUG test) [1] is a commonly used tool to assess the physical performance of orthogeriatric patients over 60 years of age. It is important for the long-term care of patients to have reproducible examinations available for an evaluation of the therapy success. Conducting physical tests such as the TUG test to objectively measure the physical segment of frailty is crucial, as recent research has revealed that simple clinical evaluation correlates poorly with objective geriatric assessment. [2,3]. The majority of individuals with numerous geriatric deficits subjectively underestimated their actual frailty in comparison to an objective assessment [2,3]. Consequently, there is a pressing need to more objectively assess the physical capacities of geriatric patients [4]. The current tools used to assess physical capacity are mainly based on standardized patient-reported questionnaires, such as the Barthel Index [5], DeMorton Mobility Index [6], or screening questionnaires, such as the sarcopenia and physical frailty screening questionnaire (SARC-F) [7]. The main disadvantages of these questionnaires are that they are very time-consuming to administer and are influenced by the subjective self-assessment and/or assessment by the caretakers. In addition, older patients tend to overestimate their physical activity [2] and patients treated in trauma surgery are often immobilized, which limits their capacity to undertake physical testing. ML-based fall detection and prevention systems are evaluated in a review by Usmani et al., with a focus on the impact of old age on increased fall risk. The frequent use of support vector machines is an often-used algorithm, and wearables for these applications are common. However, limitations arise from primarily conducting studies in controlled environments with adults, and future research directions such as energy efficiency, sensor fusion, context awareness, and wearable design are highlighted [8].

A review on the latest research trends on fall risk prediction including over 1000 studies showed that below 5% of the studies evaluated the quality of fall risk prediction models. These models used patient assessment data related to physical and cognitive function, but often did not consider post-admission factors or interventions, as well as cross-sectional blood-work data. The reporting quality was generally poor, but it has improved over the past decade. The review recommends exploring artificial intelligence and machine learning with high-dimensional data from digital hospital systems to enhance fall risk prediction in hospitals [9].

In the future, telemedicine systems will play an important role in this automation to close the gap between inpatient monitoring and outpatient care. This may help to address the unique needs of patients and their environmental contexts [10]. A user-friendly portable digital system for sarcopenia assessment, following the EWGSOP2 algorithm, has already been established by Teixeira et al. in 2022. This system not only facilitates the diagnosis and monitoring of sarcopenia but also holds potential for increasing public awareness about sarcopenia’s characteristics and risk factors [11].

For future applications, the principles and interventions set out by Petretto et al. address the potential digital paradox, where individuals who could benefit the most from telemedicine may be inadvertently excluded, particularly individuals with disabilities and the elderly. These principles encompass structural considerations, knowledge and skill requirements, and necessary adaptations, with a focus on accommodating diverse user needs. The needs and specificities of all stakeholders, including healthcare professionals and caregivers, are regarded as integral to the discussion [12].

Because of these limitations, we aimed to develop a more objective test to obtain a measure of physical performance in elderly patients by generating multifactorial data without mobility data. Second, we validated that test by comparing it to TUG test data by utilizing supervised machine learning methods [13]. It is challenging to evaluate the pertinent influencing factors holistically with reference to the individual risk, especially when evaluating multi-factorial disorders, such as physical frailty. Here, machine learning algorithms have a lot of potential to assist human judgment and enhance patient care. The long-term objective is to utilize the pilot study’s findings to create clinical decision support tools that can be linked into hospital information systems to automatically identify patients at risk.

## 2. Materials and Methods

### 2.1. Patient Recruitment

We recruited patients attending our orthogeriatric outpatient clinic, with a primary focus on osteoporosis treatment, during the period spanning December 2020 to March 2021. Our inclusion criteria encompassed individuals aged over 60 years who demonstrated independent ambulation without reliance on walking aids and exhibited no signs of mental or neurological impairments. Conversely, we excluded patients with dementia, those currently undergoing acute tumor treatment, or individuals who had sustained significant lower extremity injuries, such as fractures or joint replacements, within the preceding 6 months, to ensure the validity of the investigations, ensure the reliability of patient-reported questionnaires, and limit the impact of concurrent illnesses, as well as acute regenerative processes of the musculoskeletal system, on the laboratory values. Prior to their participation in the study, all participants provided informed consent, which encompassed the anonymized evaluation and publication of collected data. Ethical approval for the study was granted by the local ethics committee of Ludwig Maximilians University Munich (Protocol #19 177).

### 2.2. General Data Assessment

A single, properly trained investigator collected all data, including age, weight, height, BMI, body composition, blood draw, general health-related quality of life as measured by the European Quality of Life 5-dimension (EQ-5D-5L) questionnaire [14], and SARC-F [15]. To ensure data quality, we completed all surveys with the patients. A clinically approved body composition monitor was used to determine body composition regarding body fat and muscle percentages (BF511, Omron-Healthcare, Kyoto, Japan).

### 2.3. Data Collection

Data collection for each individual patient was conducted following their regular appointment at the geriatric traumatology osteoporosis outpatient clinic, typically between 9 am and 1 pm. This timing was chosen to minimize the potential impact of circadian fluctuations in the measured parameters. A single examiner conducted the data collection to ensure consistency and reduce inter-observer variability. When patients met the inclusion criteria for the study, they were provided with information about the potential study participation and given the autonomy to decide whether they wished to take part in the examinations. During data acquisition, our foremost objective was to gather a comprehensive set of parameters pertinent to physical frailty. These parameters were obtained within the confines of routine clinical practices. To uphold methodological precision, we referred to established guidelines and the pertinent literature recommendations. In particular, laboratory values from a standardized osteological screening laboratory, according to the current DVO guideline, were included as an essential component of data collection. [16] It was expanded to include the muscle markers myoglobin, LDH, and muscle-specific creatine kinase. In addition, demographic data, such as age, weight, height, BMI, were collected, and a BIA (bioelectrical impedance analysis) was used to measure body fat and muscle percentage. EQ-5D-5L was surveyed as an index for health-related quality of life. SARC-F [15] was completed with assistance given to the patients to ensure the greatest possible data quality. Patients were asked if they can lift 5 kg, walk across the room, struggle to get out of a chair, climb 10 flights of stairs, and how many times they have fallen in the previous year. Together with handgrip strength, measured using a digital dynamometer (EH101, Kuptone, London, UK) and Timed-Up-and-Go time measurements, 65 variables were collected for each patient. In shaping the parameter selection, we conducted a thorough evaluation by comparing guidelines and the current literature within the context of an expert panel, while also taking into careful consideration the available resources for data collection.

### 2.4. Timed-Up-and-Go Test

Subjects were instructed to walk from a seated position on a regular chair to a marker 3 m away, turn around, and return to the starting position in the TUG test. For all subjects, the same iPhone application (Apple Inc., Cupertino, CA) was used to record timings.

### 2.5. Clinical Laboratory Data

To minimize biochemical alterations of the blood, the samples were evaluated immediately after blood collection in the hospital’s central laboratory. An extended osteological basic laboratory [16], broadened to include muscle markers, was obtained, including sodium, potassium, glucose, creatinine clearance, creatinine, serum calcium, protein-corrected serum calcium phosphate in serum, total protein, c-reactive protein electrophoresis, albumins, beta globulins, gamma globulins, alpha-1 globulins, alpha-2 globulins alkaline phosphatase, gamma-glutamyl transferase, count of red blood cells, erythrocytes, leukocytes hematocrit, hemoglobin, average corpuscular volume mean corpuscular hemoglobin concentration, mean corpuscular hemoglobin, platelets hormone parathyroid, thyroid stimulating hormone, 25-hydroxyvitamin D3, lactate dehydrogenase, creatine-kinase, glomerular filtration rate (GFR), and myoglobin. A detailed list can be found in the supplementary data (Table S1) and on the projects GitHub repository [17].

### 2.6. Machine Learning Model Construction

The data analysis and modeling was carried out after data collection was completed using the open source programming language R (version 4.2.0), utilizing library mlr3 [18] and its dependent packages. To perform a dimensionality reduction for the machine learning algorithms, we used six different feature selection methods of the praznik package [19], each applying a threshold of 0.8 on the mutual information score (mi-score) [20] to select the most relevant variables. When the ground truth is unknown, the mi-score may be used to assess the agreement of two independent label assignment strategies on the same dataset. Comparing feature selection methods helps to make informed decisions about which method to use for specific data and objectives, considering mathematical underpinnings and trade-offs between information gain and redundancy reduction [21].

Therefore, the following six methods were selected based on their suitability for the present dataset: impurity (imp), which evaluates variables based on Gini impurity, which is used to split data in decision trees [22]; A minimum redundancy maximal relevancy filter (mrmr), which aims to minimize redundancy among selected features while maximizing their relevance to the target variable [23]; A minimal conditional mutual information maximization filter (cmim), which seeks to maximize conditional mutual information, focusing on the dependence of a feature on the target variable given the other selected features [24]; a minimal joint mutual information maximization filter (jmim), which focuses on maximizing joint mutual information, considering the mutual information of a feature with all other selected features [25]; a minimal normalized joint mutual information maximization filter (njmim), which is similar to the jmim and njmim and also maximizes joint mutual information but with the additional step of normalizing the mutual information values [26]; and a joint mutual information filter (jmi), which maximizes joint mutual information but without normalization [22]. As described, these methods differ from a mathematical point of view in how they evaluate the variables in terms of entropy, either minimizing redundancy or maximizing information gain, and whether they normalize the input data or directly use the data structure of the raw data.

The process of variable selection was followed in our analyses by training four different algorithms: the random forest algorithm [27], one generalized linear model [28], a support vector machine (SVM) [29], and an XG-Boost-algorithm [30]. During the training process, we performed resampling by five-fold internal cross-validation to increase the reliability of our models. The data were split into training and validation data in a ratio of 80/20.

Subsequently, we evaluated and compared the models with respect to their training and testing error. For this purpose, the error measures mean squared error (MSE), root mean squared error (RM0SE), and mean absolute error (MAE) were used, as a combination of metrics is often required to best assess the performance of a model [31].

Based on these results, boxplots, correlation, residual plots, and Taylor diagrams [32] were created to visualize the results.

### 2.7. Statistical Analysis

To enhance comprehensibility of the dataset, the analysis was initiated with a comprehensive descriptive statistical examination. This initial phase involved the computation of mean values and standard deviations for all numerical variables. Concurrently, categorical and binary variables were presented in terms of their respective percentage frequencies. In addition to our ML approach, a multivariate ANOVA analysis was performed to discover the optimal combination of variable extraction and algorithm selection by determining statistical differences in the training and testing errors between the utilized learners and feature selection approaches. To maximize traceability, the complete code used can be viewed in the project’s GitHub repository [17].

## 3. Results

Of the 115 eligible patients in our outpatient clinic, 103 agreed to participate in this study. In five instances, participants declined to take part in the assessments, citing that their subjective physical capacity was insufficient to complete all the tests. Additionally, seven patients declined participation due to scheduling commitments. Table 1 shows the general demographic data of these patients. See the Supplemental Materials Table S1 for a comprehensive exploratory data analysis.

### 3.1. Feature Selection Process

The cut-off of 0.8 for the mutual information score resulted in 10 selected features for each of the six methods.

**Figure 1:**
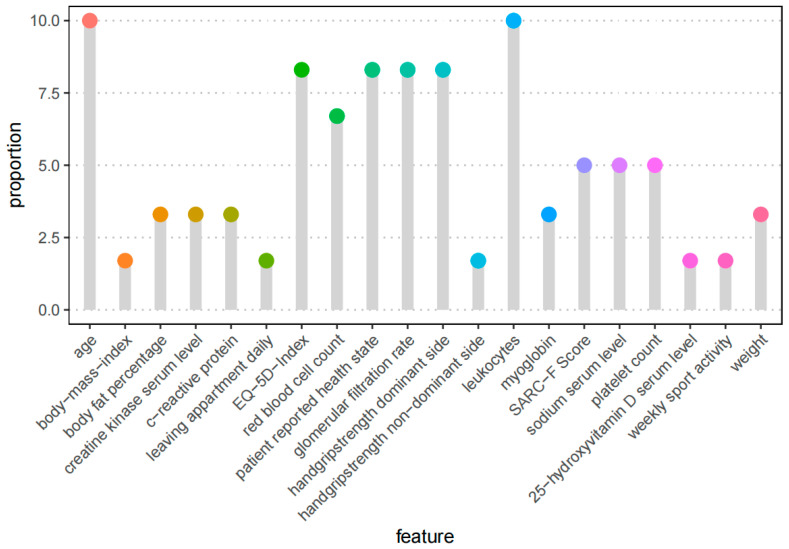
Feature frequency of the top 20 automated selected features. The process included the 6 feature selection methods described before. Each approach picked 10 features, for a total of 60, yielding a proportion of 10% if a variable is selected by all six methods and 1.6 percent if it is chosen by one method.

The evaluation of the feature frequency in our six different feature selection methods showed that age and leukocytes were the two most frequently selected variables for the regression analysis. They were selected by all six methods. By five methods, the variables EQ-5D index, GFR, grip strength of the dominant hand, and patient-reported health state were selected. The frequencies of all the selected features are shown in Figure 1 by proportion.

### 3.2. Validation of the Model

**Figure 2:**
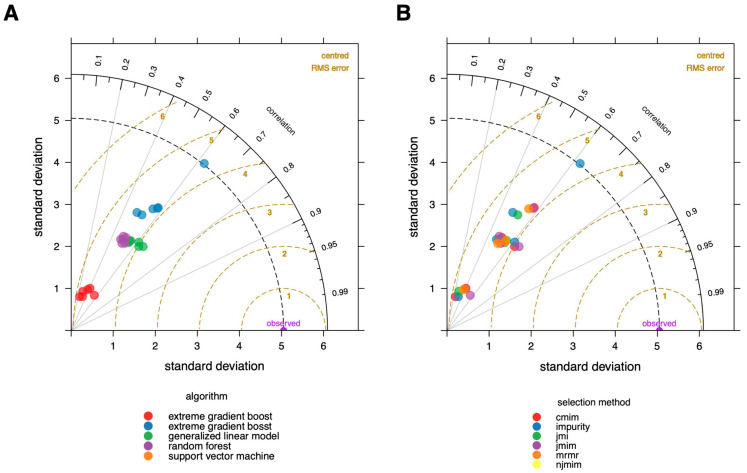
Taylor diagram of modelling results. The degree of agreement between modeled and observed behavior are visualized using three statistics: the Pearson correlation coefficient, the root-mean-square error (RMSE), and the standard deviation. (**A**) Colors correspond to the used algorithms; (**B**) colors correspond to the feature selecting methods. It is evident that the random forest algorithm is the best fit, and algorithm selection has a higher impact on the ultimate performance of the model than feature selection approaches.

To obtain an initial overview of the performance of the different models, we created a Taylor diagram [32] (Figure 2) in which the used algorithms are color-coded in A and the feature selection methods in B. This graphic, published first by Taylor in 2001 [32], aids in the comparison of several models. It measures the degree of agreement between modeled and observed behavior using three statistics: the Pearson correlation coefficient, the root mean square error (RMSE), and the standard deviation.

The Taylor diagram provides a clear summary of how the models differ in terms of performance, as assessed by the root mean square error (RMSE). The choice of algorithm clearly has a considerably bigger influence on the overall performance when compared to the feature selection method. The random forest method outperforms the other algorithms, and xgboost seems to perform the worst on our data.

To dissect the differences between the models used in more detail, we have created a summary table with three different error measures that differ, particularly in terms of their penalization of the outliers. The three testing error measures MSE, RMSE and MAE are listed in Table S2, broken down by the feature selection methods and algorithms used.

When comparing the models created using the root mean squared error (RMSE), the combination of the cmim feature selection and the random forest algorithm performed best with 3.7 s, whereas the RMSE of the xgboost is more than twice as large with 8.9 s. The MAE, representing the average of all the absolute errors, was lowest for the combination of random forest algorithm and a mrmr at 2.7 s and highest for the combination of xgboost and an njmim at 7.9 s.

**Figure 3:**
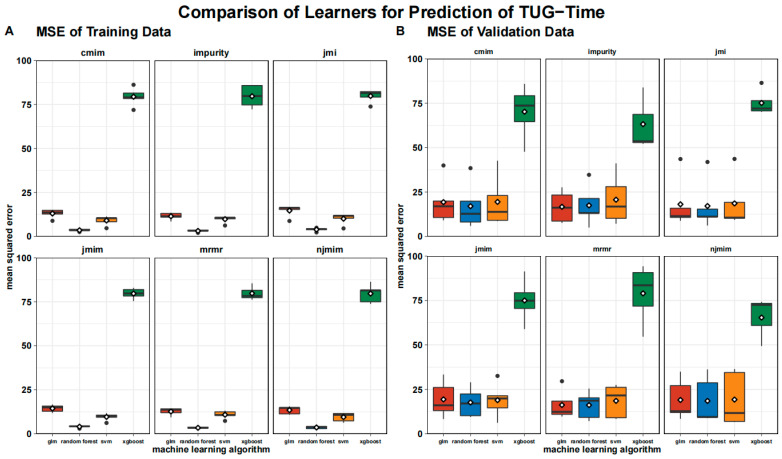
Boxplot of mean squared error of testing (**A**) and training data (**B**) grouped by algorithms and feature selection techniques. Means are shown as white rhombus, outliers are presented as black dots.

The MSE is visualized in Figure 3, where we show the MSE split into the training data and the test data. The MSE is significantly higher for the test data than for the training data across all the algorithms and feature selection methods, except for xgboost, where the training and testing errors are almost identical.

**Figure 4:**
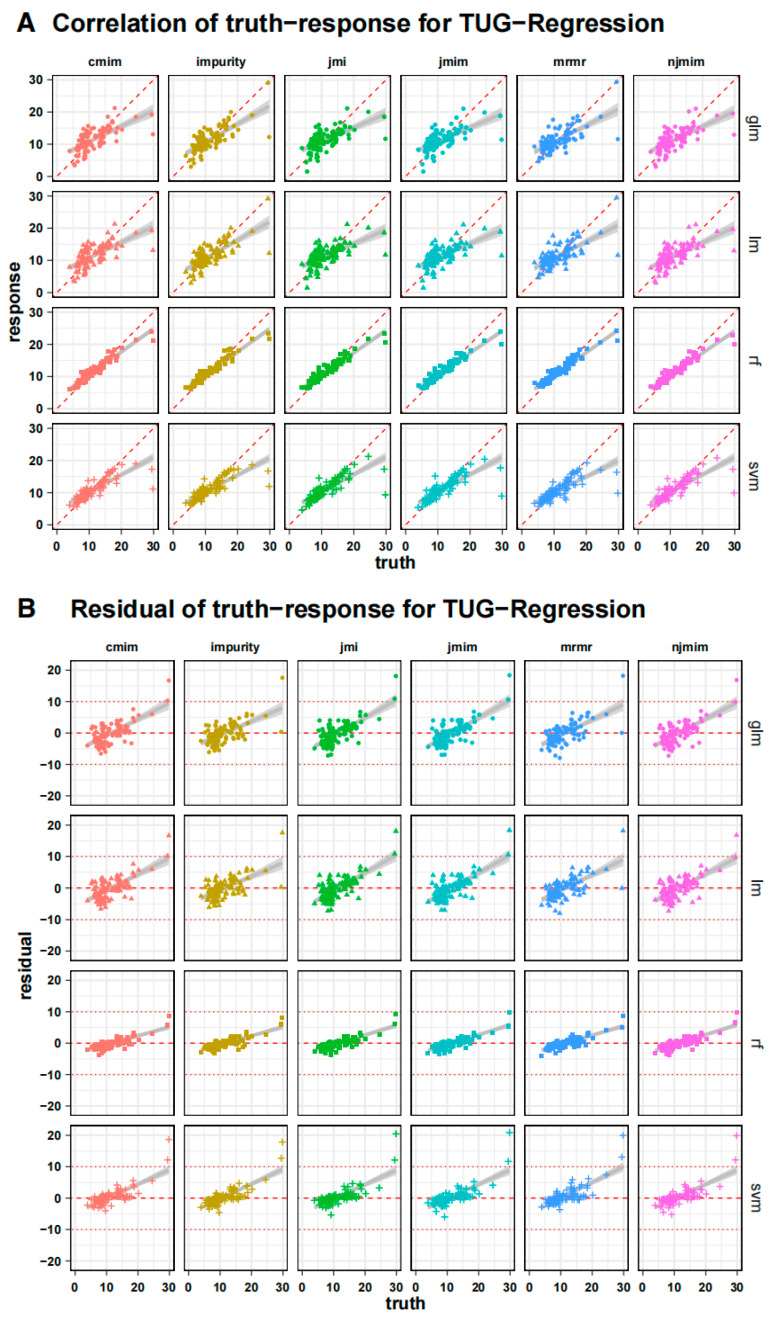
Individual training process outcomes as a correlation plot (**A**) to the subjects’ actual values after cross-validation, only showing the testing data in each instance. The dashed red line represents a perfect correlation of 1. The matching residual plot (**B**) shows a considerable increase in predicting error when the true time increases across all models. The horizontal line shows a residual value of 0 and ± 10 as reference.

Figure 4 shows the individual results of the training process as a correlation plot to the actual values of the subjects after cross-validation, with only the test data in each case. Next to it is the corresponding residual plot, which revealed a significant increase in the actual time required. This pattern can be seen in all the algorithms used, whereby it is evident that these erroneous deviations are significantly greater with longer TUG test times with the generalized linear model and the support vector machine than with the random forest algorithm. Accordingly, the random forest algorithm overestimates the slow patients less when compared to all the other algorithms. The xgboost algorithm performed worst in the training and testing processes. With respect to the feature selection methods, only a few differences can be identified.

To comprehensively assess the outcomes achieved through the implementation of machine learning techniques, a test statistic was applied to the presented results. For comparison, the models were categorized based on their respective algorithm types, followed by an ANOVA-based pairwise comparison. The ANOVA analysis, with Tukey’s multiple pairwise comparisons of the mean squared error in TUG time estimation, revealed a significant inferiority of xgboost compared to the other three algorithms (*p* < 0.001). No statistically significant disparities were observed among the remaining three algorithms, namely the random forest algorithm, generalized linear model, and support vector machine.

## 4. Discussion

Using multifactorial non-mobility data from over 100 patients, we were able to successfully develop machine learning models that predict TUG test times relatively reliably. We only used data that can be collected from bedridden patients. Our findings should help to better stratify acutely immobile patients in terms of their risk of physical frailty, allowing clinicians to make more appropriate therapeutic decisions [33]. It is crucial to bear in mind that machine learning models are founded on correlations and not causations [34]. This aspect must be considered when interpreting our results. The aim of developing these models is to provide clinical practitioners with valuable support in their assessment of frail patients, ultimately optimizing patient care.

The outcomes of our study not only advance the accuracy of TUG test time predictions but also shed light on algorithmic behavior in different patient mobility contexts. These insights are invaluable for optimizing predictive models in orthogeriatric care and have broader implications for enhancing clinical decision support systems across various healthcare domains. The achievement of a mean absolute error as low as 2.7 s underscores the potential of machine learning in refining the accuracy of TUG test time estimations. Increasing the level of accuracy is pivotal, as even small discrepancies in TUG test time predictions can have substantial clinical repercussions, affecting patient care plans and interventions [35].

Our findings reveal an important nuance in the behavior of the algorithms—the tendency to overestimate the TUG test time for quicker patients and underestimate it for slower patients. Addressing this issue in further studies is paramount to ensuring the predictive models’ clinical utility across a wide spectrum of patients with varying mobility levels.

The broader applicability of our findings extends beyond the specific context of orthogeriatric patients. The machine learning methodologies employed in this study can serve as a foundation for predictive modeling in various clinical scenarios where mobility or frailty assessment plays a pivotal role. These scenarios encompass not only fall risk estimation but also patient rehabilitation planning, resource allocation, and personalized care strategies.

The field of feature selection plays a critical role in data analysis and machine learning, aiding in the identification of relevant variables for predictive modeling [36]. Common approaches include variable filtering, which ranks variables based on their relevance to the target using predefined criteria. Other methods, such as wrapper and embedded techniques, optimize feature sets based on the performance of subsequent learning algorithms [37]. Filtering is often favored for its computational efficiency, reduced risk of overfitting, and generic applicability across various inference models. Information-theoretic measures, such as mutual information (MI) and conditional mutual information (CMI), are popular criteria for variable selection due to their model-independent nature and capacity to capture variable dependencies of arbitrary order [38]. The existing definitions of feature relevancy and redundancy fail to rigorously address interactions among variables, impeding practical feature selection methods. Discrimination power analysis is a different method for feature selection, firmly rooted in the principles of inter-class and intra-class variation, and excels in discerning the discriminatory capacity of individual features within a dataset [39]. It is particularly adept at identifying features characterized by low correlation and high discrimination, making it invaluable when dealing with complex databases comprising multiple classes and abundant training samples. DPA’s ability to balance inter-class differences and intra-class consistency ensures the selection of features that contribute significantly to predictive accuracy while reducing redundancy, which is especially important in feature extraction from multi-faceted data, such as images or shapes [40]. Since our work focuses on how multiple variables contribute information to the target of TUG test time and comparing different machine learning algorithms, our study utilized information-based methods to identify feature relevancy and redundancy in information-theoretic terms [41].

The final performance of the models was only slightly affected by the usage of various feature selection techniques. We attempted to identify the most helpful variables for machine learning using a variety of feature selection approaches, all of which were information-based, due to the high dimensionality of the dataset created by our investigations. Since there were no appreciable performance differences between the strategies and the evaluated feature selection procedures, all approaches may be approximately compared for our purposes.

Age and inflammatory parameters seem to be crucial factors for the estimation of the TUG test. To generate valuable information from the results of the feature selection methods, we tried to evaluate the frequency with which the individual variables were selected. The two variables chosen from all the selection methods, age and leukocyte count, appear to be key influencing factors for physical frailty syndrome [42]. Reviews over the past few years have shown that a high leukocyte count is a sign of systemic inflammation, illness progression, and a poor prognosis [43], and all-cause mortality can be predicted by systemic inflammation [44].

Aging is a process that happens at wildly varying speeds in various people. It appears to be a highly significant and trustworthy indicator when it comes to physical performance. This may be due to the fact that peak muscle and bone mass deterioration begins in the 20s and 30s [45]. As a result, the age attained provides critical information on how much of the musculoskeletal structures remain. A limiting note here is that muscle mass alone is not a determinant of preserved function, and degradation is subject to interindividual variation. If chronological age was extended to include biological age, the accuracy of the results achieved would most likely increase, since it is well known that biological methods of determining age are even more consistent with functional resilience than chronological age [46].

In addition, existing analyses on composite biomarker predictors for biological age also found that, for example, CRP and hemoglobin serum levels are meaningful predictors of biological age, which were also deemed relevant in our analyses [47].

The two described variables were followed in terms of importance by self-assessed health status, GFR, EQ-5D index, and handgrip strength of the dominant hand. These variables are already used in existing scores such as the Fried Frailty Scale or functional age estimators, among others. The fact that we were able to reproduce these results underlines the reliability of the factors found.

The most commonly used tool, the frailty index [48] offers the advantage that only external, physical appearance has to be assessed, and no aperitive diagnostics are necessary. Its only drawback is that the decision is made solely based on a personal assessment of external factors. Because we intended to generate the highest level of objectivity and reliability, we opted against using the Frailty Index in our investigations. Recent research in constrained patient groups has demonstrated that the TUG test and handgrip strength are also excellent tools for estimating mortality risk. This implies that the TUG test could function as a reliable gauge of biological age. Additional functional and molecular level research is required to test this theory [49].

The random forest algorithm yields the best results in the estimation of the TUG test in the utilized dataset. The algorithm we used for variable selection appears to play only a minor role in the final performance. While all algorithms, except the xgboost, start to overestimate the TUG test time of relatively fast patients and underestimate the TUG test time of slow patients, which should be improved in the further development of the algorithms, only the xgboost dramatically underestimates the time of all subjects. Our pilot study thus showed that it is possible to create relatively reliable models for estimating the TUG test time without directly using mobility data. Statements about the most important influencing factors in the utilized models could also be made, thus fulfilling the demand for explainable AI in clinical decision support systems [50].

In the present study, only classic supervised machine learning algorithms were used due to the fact that classic AI algorithms perform similarly well to deep learning approaches with the present small number of subjects, and the explainability of the algorithms used is significantly better than with deep learning approaches [51]. This is because deep learning approaches, such as deep neural networks, obscure the decision cut-offs, which makes it much more difficult to understand the decision-making process. Considering that the random forest algorithm just had a mean absolute error of 2.7 s and the utilized variables are solely based on non-mobility data, this can be considered a good result, especially when considering that many of the patients to be evaluated need a TUG test time of more than 20 s, which means that the mean absolute error is over 10%. If an imprecision of more than 10% must be expected when estimating functional outcomes, its use as a valid diagnostic tool is limited. Since the models we have developed are mainly intended to be used for risk stratification, the deviation does not have very serious direct consequences.

It should be highlighted that the subject we address, estimating mobility using non-mobility data, is dependent on complicated linkages that are challenging to answer more precisely.

Larger differences could be found between the used algorithms when compared to the feature selection methods. The combination of the impurity filter and the tree-based random forest algorithm was the best-performing algorithm in our evaluations. The reason for this could be that the random forest algorithm achieves good results, especially with diverse data structures. It should be noted that the training error of the random forest algorithm is significantly lower, when compared to the other algorithms, which leads to the risk of overfitting [52] and thus limits the generalizability. The SVM, for example, has a higher validation error in our evaluations, but at the same time, the training error deviates less from the validation error, which suggests a better transferability of the results to a larger patient population.

For very slower TUG pace, the predictions of our model become significantly less accurate. This is since we have a few subjects with very extreme TUG test times in the training data, and, at the same time, the parameters used take on very extreme values, which makes it difficult for the algorithm to make precise predictions with the relatively limited number of individual datasets. Furthermore, it is possible that additional factors, such as current motivation or other factors that we did not collect, play a relevant role in the longer TUG test times.

## 5. Summary

- Multifactorial non-mobility data from over 100 patients enabled the development of reliable machine learning models for predicting TUG (Time-Up-and-Go) test times in bedridden patients.
- The choice of feature selection techniques minimally impacted the final model performance.
- Age and inflammatory parameters, particularly leukocyte count, emerged as crucial factors in TUG estimation, indicative of systemic inflammation and mortality risk.
- Biological age, incorporating factors such as CRP and hemoglobin levels, correlated with the TUG outcomes.
- Variables such as self-assessed health, GFR, EQ-5D index, and handgrip strength were identified as influential, aligning with existing frailty assessment tools.
- The random forest algorithm outperformed the other ML algorithms in TUG estimation
- The study achieved a mean absolute error of 2.7 s in TUG estimation, though limitations existed for TUG test times over 20 s, potentially due to limited extreme data and uncollected factors such as motivation.
- Estimating mobility from non-mobility data involves complex relationships, posing challenges.
- The impurity filter combined with the random forest algorithm showed the best performance, although overfitting risk and lower validation errors were noted.

## 6. Limitations

The number of subjects included in the analysis is relatively low for a machine learning approach. However, it is only an exploratory pilot study investigating the special patient population of orthogeriatric patients. Another limitation within the confines of our preliminary investigation pertains to its monocentric study design. This particular design imposes constraints on the extrapolation of findings, particularly in the context of applying machine learning algorithms, due to the inability to aggregate structural attributes specific to the study center across multiple centers. Therefore, the results should only serve as a basis for further studies. The measures proposed here, which appear to be relevant for assessing physical frailty, should be evaluated in larger-scale, ideally multicenter research.

Since our study was designed in a single-stage, single-center setting, during the model creation, an internal five-fold cross-validation was conducted to create more generalizability. We recognize the importance of prospective validation to corroborate the robustness of our findings. Future research initiatives should focus on validating our predictive models in independent cohorts of orthogeriatric patients to assess their generalizability and clinical applicability. We made the models open-source to enable validation across patient populations.

The very-slow-walking patients were especially difficult to estimate correctly. According to our findings, the slower the patients get, the more difficult the correct prediction becomes. In the future, investigations of only these slower patients will be necessary to better understand the underlying relationships and thus be able to make better assessments.

Machine learning studies are always based on correlation analyses, which take a closer look at the data structure. Therefore, the results must not be considered causal, but only represent a possibility to understand the correlations and patterns in the data and to be able to draw clinically relevant correlations from them, which are not necessarily subject to direct causalities.

No sample size calculation was performed for our study as it was conducted as a pilot investigation. The predetermined target sample size of 100 individuals was selected primarily to facilitate a fundamental correlation analysis.

## 7. Conclusions

Our results demonstrate that non-mobility data can be used effectively to forecast the time required for the TUG test in orthogeriatric patients using machine learning models, although the more time patients needed, the less accurate the predictions became.

This is a building block to automate the detection of patients at risk and to create the possibility of also objectively assessing immobilized patients regarding their physical capacity. Statements regarding the most influential aspects of the employed models could also be made, thus meeting the requirement for explainable AI in medicine and at the same time gaining new insights into physical frailty and related factors. Future research is required to confirm our findings and adopt clinical decision support systems based on the developed algorithms.

## Figures and Tables

**Table 1:** Demographic patient data (*n* = 103, IQR = inter-quartal range).

Variable	N	Median	IQR
Age	103	76	(71, 80)
Handgrip strength		22.4	(18.8, 25.2)
TUG test time		9.5	(8.0, 13.8)
Weight		64	(58, 70)
Height		162	(158, 166)
BMI		24.4	(21.7, 25.9)

## Data Availability

The corresponding author can provide the data described in this study upon request. Due to laws governing data protection and privacy, the data are not accessible to the general public.

## Supplementary Materials

The following supporting information can be downloaded at: https://www.mdpi.com/article/10.3390/geriatrics8050099/s1, Table S1: Explanatory Data Analysis of complete variable set presented in mean and inter-quartile range (IQR) in parentheses, Table S2: Results of model evaluation, ordered in descending order.
