# Supplementary material for: Development of a Machine Learning-Based Model to Predict Timed-Up-and-Go Test in Older Adults
Source: Geriatrics (Basel). 2023 Oct 7;8(5):99. doi: 10.3390/geriatrics8050099 (PMC10606325; doi:10.3390/geriatrics8050099)
Supplement: Supplementary file 1 [file geriatrics-08-00099-s001.zip › geriatrics-2567466-supplementary.pdf]

geriatrics

MDPI

Supplementary Materials

Development of a Machine Learning Based Model to predict Timed up and Go Test in Older Adults

Table S1. Explanatory Data Analysis of complete variable set presented in mean and inter quartile range (IQR) in parentheses.

Characteristic	N	N = 103[1]
SPPB Score	103	10 (7, 12)
Handgrip strength dominant hand	103	22.4 (18.8, 25.2)
Handgrip strength non dominant hand	103	18.9 (15.3, 22.6)
TUG-Time	103	9.5 (8.0, 13.8)
Sodium	103	139.64 (138.00, 141.00)
Potassium	103	4.60 (4.50, 4.80)
Glucose	103	89 (86, 97)
GFR	103	77 (69, 86)
Calcium	103	2.42 (2.36, 2.48)
Calcium corr.	103	2.46 (2.40, 2.50)
Phosphate	103	3.70 (3.40, 4.00)
C-reactive-protein	103	0.10 (0.10, 0.20)
Total protein	103	6.90 (6.70, 7.10)
Gamma gluteryl transferase	103	23 (16, 32)
Alkaline phosphate	103	68 (58, 77)

Characteristic	N	N = 103[1]
**Leukocytes**	103	6.08 (5.44, 7.42)
**Erythrocytes**	103	4.48 (4.25, 4.71)
**Hemoglobin**	103	13.42 (13.00, 14.00)
**Hematocrit**	103	388 (295, 422)
**MCV**	103	90.3 (88.6, 92.6)
**MCH**	103	30.11 (29.55, 30.90)
**MCHC**	103	33.30 (32.60, 33.85)
**Thrombocytes**	103	250 (222, 275)
**TSH**	103	1.31 (0.90, 1.62)
**Parathormone**	103	46 (37, 51)
**Vitamin D3**	103	45 (39, 49)
**LDH**	103	214 (198, 228)
**Creatine kinase**	103	103 (75, 123)
**Creatinine**	103	0.80 (0.70, 0.90)
**Myoglobin**	103	41 (30, 54)
**Bone mineral density femoral neck**	103	484 (1, 600)
**Bone mineral density L1**	103	1 (1, 804)
**Time for 4m**	103	3.10 (2.60, 4.30)
**Time for 5x sit to stand**	103	12.2 (10.3, 14.7)

**Characteristic**	**N**	**N = 103**[1]
**Age**	103	76 (71, 80)
**Weight**	103	64 (58, 70)
**Height**	103	162 (158, 166)
**BMI**	103	24.4 (21.7, 25.9)
**Body fat percentage**	103	32 (27, 36)
**Visceral fat percentage**	103	8.00 (6.00, 10.00)
**Muscle percentage**	103	28.7 (27.0, 31.1)
**Resting metabolism kcal**	103	1,352 (1,284, 1,414)
**Calf circumference**	103	34.86 (33.50, 36.50)
**SARC-F Score**	103	
0		51 (50%)
1		23 (22%)
2		7 (6.8%)
3		8 (7.8%)
4		8 (7.8%)
5		6 (5.8%)
**Self-estimated health state**	103	70 (60, 80)
**Age menopause**	100	48.0 (46.0, 50.0)
**Daily leaving apartment**	103	83 (81%)

**Characteristic**	**N**	**N = 103**[1]
**Weekly sports**	103	66 (64%)
**smoking**	103	11 (11%)
**Self-sustaining**	103	87 (84%)
**EQ5D-Index**	103	763 (1, 871)
**Falls within last year**	103	45 (44%)
**Prefrailty**	103	37 (36%)
**Osteoporosis**	103	56 (54%)

[1] Median (IQR); n (%)

Table S2. Results of model evaluation, ordered in descending order.

**Selection Method**	**Type of learner**	**Mean squared error (MSE)**	**Root mean squared error (RMSE)**	**Mean absolute error (MAE)**
cmim	random forest	15.822	3.728	2.719
mrmr	random forest	16.155	3.916	2.659
mrmr	glm	16.211	3.935	2.842
impurity	glm	16.662	3.959	2.850
cmim	random forest	17.005	3.899	2.813
jmi	random forest	17.103	3.903	2.913
impurity	random forest	17.447	3.997	2.854
jmim	random forest	17.640	4.106	2.980
jmi	glm	18.073	4.040	3.082
njmim	random forest	18.508	4.094	2.931
mrmr	svm	18.546	4.182	2.828
jmi	svm	18.577	4.096	2.893
jmim	svm	18.943	4.222	3.005
njmim	glm	19.052	4.211	3.282

Selection Method	Type of learner	Mean squared error (MSE)	Root mean squared error (RMSE)	Mean absolute error (MAE)
njmim	svm	19.259	4.111	2.972
cmim	glm	19.292	4.233	3.162
jmim	glm	19.376	4.277	3.282
cmim	svm	19.438	4.199	3.116
impurity	svm	20.655	4.334	3.124
jmim	xgboost	80.879	8.951	7.669
cmim	xgboost	81.367	8.911	7.680
jmi	xgboost	83.141	9.075	7.755
mrmr	xgboost	83.558	9.092	7.839
njmim	xgboost	85.169	9.118	7.896
